# Nerve Ultrasound in Patients With Friedreich Ataxia

**DOI:** 10.1002/mus.70091

**Published:** 2025-12-04

**Authors:** Katharina Kneer, Jan‐Hendrik Stahl, Natalie Winter, Julia Wittlinger, Stephanie Männlin, Toghrul Gasimli, Ludger Schöls, Zofia Fleszar, Stefanie Hayer, Alexander Grimm

**Affiliations:** ^1^ Department of Neurology and Epileptology Eberhard‐Karls University of Tuebingen Germany; ^2^ Hertie Institute for Clinical Brain Research, Eberhard‐Karls University Tübingen Tübingen Germany; ^3^ Department of Neurodegenerative Diseases Center of Neurology, University of Tuebingen Tuebingen Germany; ^4^ Institute of Molecular and Human Genetics, Charité University Berlin Berlin Germany

**Keywords:** Friedreich ataxia, nerve ultrasound, neurodegenerative disease, peripheral neuropathy

## Abstract

**Introduction/Aims:**

Nerve enlargement has been reported in patients with Friedreich ataxia (FRDA). The underlying cause remains unclear, and both inflammatory processes and dysmyelination have been suggested as potential mechanisms. This study was aimed at assessing nerve morphology with high‐resolution ultrasound, to identify and describe patterns of nerve pathology.

**Methods:**

Patients with genetically confirmed FRDA were included, clinically examined, and assessed with high‐resolution ultrasound. Cross‐sectional area measurements were compared with previously published normal values.

**Results:**

Among 20 included patients, ultrasound abnormalities were found in 17 (85%). Nerve enlargement of the median and/or ulnar nerve in the proximal nerve segments was observed in 45% of patients and was statistically significant compared to published normal values. Lower extremity nerve enlargement was observed in 20% of patients. Eight patients did not show any nerve enlargement. Diminished cross‐sectional area, indicative of nerve atrophy, was present, particularly in the distal segments of the ulnar nerve (in 25% of patients, *p* < 0.05) and the tibial nerve (popliteal fossa in 30% of patients, medial malleolus in 30% of patients, both *p* < 0.05). Nerve enlargement showed no significant correlations with disease severity or age of disease onset.

**Discussion:**

Peripheral nerve morphology in FRDA is variable and includes not only nerve enlargement but also nerve atrophy, thus reflecting complex segmental pathology beyond axonal degeneration.

## 1 Introduction

Although most demyelinating diseases lead to increased cross‐sectional area (CSA), as measured with high‐resolution ultrasound (HRUS), CSA in axonal neuropathies is either normal or enlarged, depending on the cause of the neuropathy, such as vasculitis, sarcoidosis, or type 2 diabetes mellitus [1, 2, 3]. In contrast, sensory neuronopathies typically result in diminished CSA, as seen in cerebellar ataxia, neuropathy, and vestibular areflexia syndrome [4].

In FRDA, a homozygous guanine‐adenine‐adenine (GAA) trinucleotide repeat expansion leads to a transcriptional defect in the *FXN* gene, thus resulting in a significant reduction of the mitochondrial protein frataxin [5]. This genetic defect results in developmental rather than progressive neurodegeneration affecting the spinocerebellar pathways, dorsal root ganglia, posterior columns, corticospinal tracts, and sensory neuropathy [6, 7, 8].

Electrophysiological examinations in patients with FRDA have indicated sensory axonal neuropathy, whereas HRUS studies have described nerve enlargement, predominantly in proximal nerves of the upper extremity, such as the median nerve [9].

A missense mutation in *FXN* is known to cause dysmyelination, and consequently axonal atrophy as well as nerve hypertrophy, as seen in other peripheral neuropathies [8, 10]. In contrast, in vitro studies have identified a satellite cell disturbance associated with inflammatory‐mediated destruction, primarily of dorsal root ganglia cells, as observed in neuronophagia [8, 11, 12]. Therefore, the exact cause of this peripheral nerve pathology—inflammation or dysmyelination—remains unknown. From a clinical viewpoint, studies have suggested a correlation between nerve enlargement and the extent of clinical impairment [13].

Previous HRUS studies are limited by small sample sizes, which introduce uncertainty regarding the generalizability and reproducibility of the reported findings.

The aim of this study was to systematically evaluate peripheral nerve morphology with HRUS, with a focus on identifying patterns of nerve pathology.

## 2 Methods

### 2.1 Cohort

Between May 2015 and December 2017, patients with FRDA were evaluated in our specialized ataxia outpatient clinic. Clinical examination and additional electrophysiological assessments, including nerve conduction studies (NCS) and HRUS, were conducted and retrospectively analyzed. The study was approved by the local ethics committee of the University of Tübingen (702/2015BO2) and conformed to the World Medical Association's Declaration of Helsinki. All examinations were conducted after informed consent was provided by all participants.

This study excluded patients with a history of harmful alcohol consumption, severe vitamin deficiency (vitamin B1, B6, B9, B12), confirmed by abnormal laboratory blood test results, or toxin exposure.

### 2.2 Clinical Assessment and Electrophysiology

The clinical assessment involved a standardized neurological examination performed by a movement disorder specialist (ZF, SH, and LS) using the Scale for the Assessment and Rating of Ataxia (SARA) [14].

Clinical signs of sensory neuropathy, assessed in all patients, included diminished or absent deep tendon reflexes, impaired proprioception, paresthesia, dysesthesia, and distal hypoesthesia.

Nerve conduction studies were performed in patients who provided informed consent, under standard conditions according to published data [15], with a Dantec Keypoint G4 workstation (Natus Medical Inc., Middleton, Wisconsin, USA). Motor nerve conduction studies included the distal motor latency, compound muscle action potential (CMAP) amplitude, and motor nerve conduction velocity (NCV) of the ulnar or median nerves in the upper extremity, and the tibial and fibular nerves in the lower extremity. F‐wave latency was tested for the median or ulnar nerves in the upper extremity and the tibial nerve in the lower extremity. Sensory nerve action potentials were assessed for the sural and ulnar nerves, with amplitudes measured from baseline to peak.

In all patients, motor nerves were tested alternately on both sides; for example, the left side for the ulnar and tibial nerves, and the right side for the fibular nerve. Sensory nerves were tested on the right side.

Neuropathy was defined as demyelinating if the NCV was slower than 75% or the distal latency was longer than 130% of normal values, and was defined as axonal if the amplitudes decreased with only a mild decrease in NCV or a mild increase in distal latency, according to Preston and Shapiro [16] (comparable to the guidelines of the European Federation of Neurological Societies and the Peripheral Nerve Society for chronic inflammatory demyelinating polyneuropathy [17]).

Additional demyelinating features included partial conduction blocks, characterized by a focal decrease in CMAP amplitude and in area by > 50% between distal and proximal stimulation sites, without temporal dispersion. Complete conduction blocks were identified according to the absence of a CMAP under proximal stimulation despite a normal distal response. Temporal dispersion was defined by a prolonged CMAP duration with diminished amplitude but preserved area [18]. Published reference values were used [19].

### 2.3 High‐Resolution Nerve Ultrasound

High‐resolution B‐mode ultrasound was performed according to a previously published protocol [20, 21] with a high‐resolution probe (range 12–18 MHz, linear transducer with 46 mm depth, Mindray T7, Ultrasound systems, Darmstadt, Germany). The “NERVE” preset was used for all participants, with consistent image amplification (80 G “Gain”). The preset corresponded to a maximum frequency of 18 Hz. Basic settings remained unchanged during the entire examination, except for focus adjustments.

The examination, conducted by experienced sonographers, assessed the CSA of peripheral nerves and the diameters of cervical roots. CSA and nerve diameter were compared with previously published normal values [22, 23, 24]. High‐resolution nerve ultrasound was performed on the right side, including the median, ulnar, radial, tibial, fibular, sural, and vagus nerves, as well as the C5 and C6 nerve roots. Each nerve was systematically evaluated along its entire course, and CSA measurements were obtained at predefined anatomical sites [4]: the median nerve at the mid‐forearm, cubital fossa, and mid‐upper arm; the ulnar nerve at the mid‐forearm and mid‐upper arm; the superficial radial nerve proximal to the forearm deep fascia; the tibial nerve at the popliteal fossa and medial malleolus; the fibular nerve at the popliteal fossa and the superficial fibular nerve; the sural nerve adjacent to the Achilles tendon; and the vagus nerve at the carotid triangle. Additionally, a longitudinal assessment of the C5 and C6 nerve roots was conducted (Figure S1).

Nerve enlargement in anatomic nerve entrapment sites, such as the carpal tunnel or the cubital tunnel, was excluded.

Nerve echogenicity was assessed subjectively by the sonographer, through comparison of the nerve echogenicity with that in surrounding tissue, including vessels, muscle, and subcutaneous fat.

### 2.4 Statistical Analysis

All analyses were conducted in SPSS version 24 (IBM, Armonk, NY, USA). Mean ± standard deviation (SD) is reported for normally distributed results, whereas the interquartile range (IQR) is reported for results that were not normally distributed.

Statistical significance was assessed with the independent t‐test for normally distributed results or the Mann–Whitney U‐test for non‐normally distributed data. The threshold for statistical significance was set to *p* ≤ 0.05. Pearson's test was used to determine correlations between variables for normally distributed data and Spearman correlation testing for non‐normally distributed data.

## 3 Results

### 3.1 Cohort

Twenty patients with FRDA were included in this study (patient characteristics summarized in Table 1). The mean age was 41.6 years (IQR: 34–64), and the mean disease onset occurred at 17 years (IQR: 10.5–22). Three patients with diabetes mellitus were included in this study, and all other patients showed no abnormal oral glucose tolerance test findings and had normal HbA1c levels.

**TABLE 1:** Baseline characteristics.

Patient	Sex	Age in years	Age at disease onset in years	Repeat length	Sensory neuropathy	SARA score	Other system involvement
1	M	40	14	N/A	Yes	32	CM
2	M	24	3	700/1100	Yes	27	CM, DMT2
3	M	38	16	250/1090	Yes	26.5	DMT2
4	F	27	12	1000/1000	Yes	13.5	None
5	F	40	15	570/1200	Yes	27.5	CM
6	F	79	43	N/A	Yes	27	None
7	M	64	46	83/500	Yes	15	None
8	F	49	20	595/730	Yes	32	DMT2
9	F	36	10	1000/1000	Yes	30	None
10	F	30	11	650/650	Yes	23.5	None
11	M	21	5	1000/1000	Yes	28	CM
12	M	34	14	533/533	Yes	29.5	None
13	F	34	8	N/A	Yes	38	None
14	M	59	13	N/A	Yes	40	CM
15	M	66	31	250/900	Yes	29.5	None
16	F	46	15	N/A	Yes	28	None
17	F	53	30	N/A	Yes	20	None
18	F	36	24	N/A	Yes	15	None
19	M	22	7	500/780	Yes	31.5	CM
20	F	34	14	N/A	Yes	14	None

Abbreviations: CM = cardiomyopathy, DMT2 = diabetes mellitus type 2F = female, M = male, N/A = not available, SARA = scale for the assessment and rating of ataxia.

### 3.2 Clinical Assessment and Electrophysiology

All 20 included patients showed clinical signs of sensory neuropathy with absent muscle stretch reflexes and impaired proprioception. Of these, 13 patients were investigated with NCS in our facility. Seven patients declined to consent to electrophysiological testing. Details of the electrophysiological measurements are summarized in Table 2.

**TABLE 2:** Nerve conduction study data.

Patient	UN DML [ms]	UN CMAP [mV]	UN NCV [m/s]	UN F‐wave lat. [ms]	TN DML [ms]	TN CMAP [mV]	TN NCV [m/s]	TN F‐wave lat [ms]	SNAP amp in μV
Norm values	3.2	4.0	50	31.0	5.1	5.1	40	Height‐dependent^a^	SN 3.8
1	ND	ND	ND	ND	ND	ND	ND	ND	ND
2	**4.3**	10.8	58	**35.9**	5	9.9	**32**	**71.4**	**NR UE&LE**
3	2.6	13.9	64	32	ND	ND	ND	ND	**NR UE&LE**
4	2.9	19.5	57	29.6	3.8	28.2	40	53.4	**NR UE&LE**
5	ND	ND	ND	ND	4.7	28	28	56.5	**NR UE&LE**
6	ND	ND	ND	ND	ND	ND	ND	ND	ND
7	ND	ND	ND	ND	4.9	29.5	46	57.5	SN 5.1
8	3.9	5.4	48	28.8	3.8	15.3	41	47.3	**NR UE&LE**
9	2.4	17	43	**32**	5.4	14.2	30	**63.7**	**NR UE&LE**
10	2.4	16	65	27.5	4.3	31.3	45	47.4	**NR UE&LE**
11	3.2	17	52	26	3.1	13.6	43	44.8	**NR UE&LE**
12	ND	ND	ND	ND	ND	ND	ND	ND	ND
13	3.3	11.7	48	31	5.1	14.5	35	52.4	**NR UE&LE**
14	ND	ND	ND	ND	ND	ND	ND	ND	ND
15	ND	ND	ND	ND	4.9	14	41	57.4	**NR UE&LE**
16	ND	ND	ND	ND	ND	ND	ND	ND	ND
17	3.0	19.9	68	24.7	4.9	34.6	41	47.2	**NR UE&LE**
18	ND	ND	ND	ND	ND	ND	ND	ND	ND
19	ND	ND	ND	ND	ND	ND	ND	ND	ND
20	ND	ND	ND	ND	4.3	22.5	49	51.8	SN **2.4**

*Note*: Abnormal values are highlighted in bold.

Abbreviations: amp = amplitude, cm = centimeter, CMAP = compound muscle potential amplitudes, DML = distal motor latency, lat = latency, LE = lower extremitym/s = meters per second, ms = millisecond, mV = millivolt, NCV = nerve conduction velocity, ND = not done, NR = no response, SN = sural nerve, SNAP = sensory nerve action potential sural and ulnar nerve (baseline‐to‐peak), TN = tibial nerve, UE = upper extremity, UN = ulnar nerve, μV = microvolt.

^a^ F‐Wave reference values: height > 178 cm: 63.3 ms, height < 178 cm: 58.0 ms.

Twelve patients demonstrated sensory neuropathy on NCS, and no abnormalities were detected in CMAP. One patient with late‐onset FRDA demonstrated clinical signs of sensory neuropathy but showed no abnormalities on NCS. Two patients showed slightly prolonged F‐wave latencies (< 130% the upper limit of normal) as well as mildly diminished NCV (< 75% of normal) in the upper and lower extremities (Table 2). In all other patients, the F‐wave latencies were unremarkable. No patients showed any electrophysiological signs of demyelination.

No significant differences were observed between patients with or without diabetes mellitus, including clinical neurological presentation and NCS data. No abnormal CMAP amplitudes were detected.

### 3.3 High‐Resolution Nerve Ultrasound

HRUS was performed in all 20 patients and ultrasound abnormalities were found in 17 (85%). Details of the sonographic findings are summarized in Table 3.

**TABLE 3:** High‐resolution ultrasound results of Friedreich ataxia patients.

Nerve CSA (mm^2^)^a^																						
	1	2	3	4	5	6	7	8	9	10	11	12	13	14	15	16	17	18	19	20	Median CSA [IQR]	*p*‐value
MN forearm (5.9–10)	**10**	6	8	**11**	7	7	8	8	8	6	8	7	*4*	8	8	6	6	8	6	7	7.0 [2.0]	0.6 U 0.1 L
MN cubital fossa (12.5)	**17**	**19**	7	11	7	9	8	9	9	7	11	12	6	6	8	7	7	6	10	7	8.0 [3.5]	0.2
MN upper arm (7.5–12)	**39**	**23**	**29**	**15**	9	9	**12**	10	8	8	9	**12**	9	*6*	11	**14**	9	9	**14**	*7*	9.5 [5.0]	< 0.001 U 0.6 L
UN forearm (4.8–8.5)	**11**	6	7	6	6	5	7	6	6	*3*	6	*4*	*3*	*3*	6	6	*4*	7	7	7	6.0 [2.5]	0.9 U < 0.001 L
UN upper arm (5.1–9.5)	**25**	**18**	**15**	**11**	**11**	9	8	9	7	9	7	**10**	9	7	9	9	5	9	**11**	7	9.0 [3.5]	< 0.001 U
TN medial malleolus (7.7–14)	*6*	*7*	9	10	9	9	8	8	*5*	*6*	9	12	**16**	8	*5*	*7*	11	10	10	11	9.0 [3.0]	0.1 U 0.01 L
TN popliteal (17.5–33 f,17.5–36 m)	*15*	23	35	30	31	28	32	25	*15*	18	24	22	*14*	*15*	**67**	*13*	27	*16*	26	26	24.5 [13.5]	0.1 U < 0.001 L
Superficial FN (3.5)	1	1	2	2	2	1	2	1	1	1	2	3	3	1	1	2	1	**4**	1	2	1.5 [1.0]	0.2
FN popliteal (6.6–11.5)	9	7	11	8	11	11	10	9	*4*	*5*	8	**16**	8	8	11	8	10	7	9	7	8.5 [3.0]	0.7 U 0.1 L
Nerve root C5 (Diameter in mm, 2.9 mm)	2.30	2.3	3	2	3	2.4	3	2	2	2.6	2.2	3	2.8	2	2.2	2.2	2.1	2.2	2.3	2.5	2.4 [0.4]	0.9
Nerve root C6 (Diameter in mm, 4.2 mm)	3.5	3	4	4	3	3.7	3	3	3	2.6	2.9	3	4	3	2.4	2.4	2.7	3	3.9	2.7	3.0 [1.0]	0.18
Vagus nerve (3.5)	2	2	2	1	2	2	3	2	3	1	2	1	2	1	2	2	3	2	3	3	2.0 [1.0]	0.1
Superficial RN (3)	2	2	2	2	2	1	2	1	1	1	2	2	1	1	2	1	1	1	2	2	2.0 [1.0]	0.8
SN (3.5)	**4**	3	2	3	3	1	1	1	1	1	3	2	2	1	3	2	2	2	3	2	2.0 [1.0]	0.1

*Note*: Abnormal nerve enlargement is highlighted in bold, abnormal nerve atrophy in italics.

Abbreviations: CSA: cross‐sectional area, f: female, FDRA: Friedreich‐Ataxia, FN = fibular nerve, IQR: interquartile range, L: lower boundary value, m: male, mm: millimeter, mm^2^: square millimeter, MN = median nerve, n: number, RN = radial nerve, SN = sural nerveTN = tibial nerve, U: upper boundary value, UN = ulnar nerve.

^a^ Normal values are in parentheses for each nerve segment in mm^2^.

Nerve enlargement of the median and/or ulnar nerves in the upper arm was observed in 9 of 20 patients (45%), two (10%) of whom also exhibited nerve enlargement in the forearm and cubital fossa, which was not statistically significant. The remaining 11 patients (55%) had CSA values that were within upper normal limits.

Nerve enlargement in the lower extremities, including the tibial, fibular and sural nerves, was identified in 4 of 20 patients (20%) but was not statistically significant. Of these, two patients also demonstrated CSA enlargement of the median and/or ulnar nerve in the upper extremity, whereas the remaining two patients showed no CSA changes in the upper limb nerves. Overall, no correlation was found between disease duration or severity and the extent of nerve enlargement (*r* = 0.1).

**FIGURE 1:**
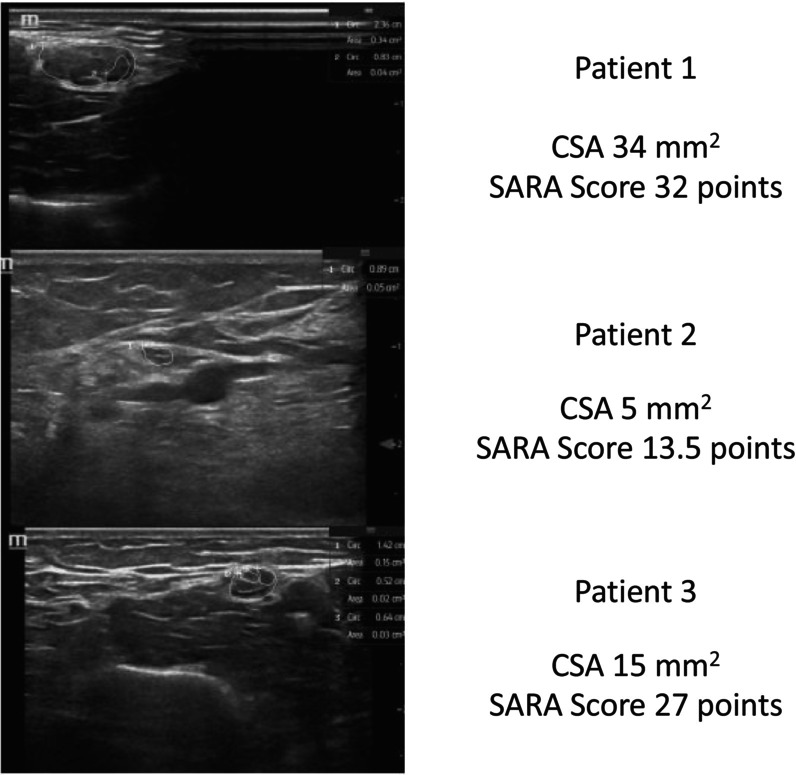
Comparison of high‐resolution ultrasound of the median nerve in the upper arm between 3 patients.

Beyond nerve enlargement, diminished CSA indicative of atrophy was observed in several nerve segments, in which the nerve itself appeared hypoechoic (Figure 1). For the upper extremity, two patients showed diminished CSA in the proximal segments of the median and ulnar nerves. A statistically significant decrease in the CSA of the ulnar nerve in the forearm was found in five patients.

The tibial nerve CSA was significantly below the lower limit of normal in 6 of 20 patients (30%) at the popliteal fossa and in 6 of 20 patients (30%) at the medial malleolus. In contrast, the fibular nerve at the popliteal fossa showed diminished CSA in only 2 of 20 patients (10%), and this finding did not reach statistical significance. No patients showed affected cervical nerve roots (C5 and C6) or vagus nerves.

Diminished CSA was identified in patients with or without previously described segmental nerve enlargement.

Both, nerve enlargement and nerve atrophy occurred in seven patients (35%); nerve atrophy alone occurred in 5 (25%) and nerve enlargement alone occurred in 5 (25%).

The diabetic patients, when compared with the non‐diabetic cohort, showed neither significant differences in the nerve conduction study data nor greater CSA.

## 4 Discussion

Overall, our findings are consistent with the current literature regarding the distribution pattern of CSA enlargement in Friedreich ataxia patients [9, 13].

Nerve enlargement was observed in both the upper and lower extremities, predominantly in the proximal segments of the upper limb nerves rather than their distal portions. Nerve enlargement in the lower limb was uncommon and non‐significant. The rate of nerve enlargement abnormalities in our cohort was notably lower than observed in previous studies [9, 13] and 40% of patients did not show any nerve enlargement at all. Given the larger sample size in our study, this discrepancy suggests that findings from smaller cohorts might not be directly generalizable to broader patient populations.

Beyond nerve enlargement, diminished CSA indicative of nerve atrophy was found to affect the nerves of the upper and lower extremities, preferentially affecting the tibial nerve and the distal segment of the ulnar nerve. This atrophic pattern was present in both subgroups, i.e., patients with or without segmental nerve enlargement. Nerve enlargement and atrophy were sometimes observed within the same nerve but in different anatomical segments, thus underscoring the heterogeneous nature of peripheral nerve involvement in FRDA.

We observed no differences in the age and sex distributions between our patient cohort and previously described FRDA cohorts [9, 13]. Our electrophysiological data are in line with findings from prior studies showing a sensory axonal neuropathy in 12 of 13 examined patients [13]. In contrast to previously described FRDA cohorts, no correlation was found between disease duration or severity and the extent of nerve enlargement [13].

The underlying cause of this pleomorphic sonographic pattern remains unclear:

As previously described by Mulroy et al. [9], a primary inflammatory process might potentially account for the observed nerve enlargement. Similar to other inflammatory neuropathies, such as Guillain‐Barré syndrome or chronic inflammatory demyelinating polyneuropathy, the predominant involvement of proximal arm nerves might support this hypothesis. Cervical root involvement has been reported in more than 90% of immune‐mediated neuropathies [1, 25, 26] but was completely absent in our and previously described FRDA cohorts [9, 13]. This absence of cervical root enlargement alone should not be considered to exclude the possibility of an inflammatory component. Nerve enlargement preceding nerve atrophy, due to a disturbed blood‐nerve barrier, may be considered, as has been observed in other neurodegenerative disorders, such as amyotrophic lateral sclerosis [27].

Alternatively, dysmyelination induced by the frataxin mutation might lead to compensatory remyelination and subsequent hypertrophic remodeling, as described in other neurodegenerative diseases. Evidence from murine models has demonstrated the presence of axonal vacuoles, which might serve as a compensatory mechanism in response to ongoing cycles of degeneration and regeneration [28].

Whether these processes occur simultaneously and continuously in every nerve fascicle and segment remains unclear. Our findings suggested an asynchronous degeneration–regeneration cycle across different nerve segments.

In cases of advanced axonal loss, hypertrophic remodeling might be limited to fascicular enlargement, as previously described by Pitarokoili et al. [29]. However, whether a failed remyelination process ultimately leads to secondary inflammation or nerve atrophy remains uncertain.

In cases of significant nerve enlargement, further diagnostic steps—such as lumbar puncture, additional blood analysis, MR neurography, or, if possible, nerve biopsies to rule out any other treatable inflammatory processes indicated by albuminocytological dissociation, contrast agent uptake, or lymphocytic infiltration—might be warranted. Additionally, individual therapeutic approaches, including immunotherapy, could be considered.

Limitations of this study include the absence of electrophysiological data in seven patients and the lack of bilateral electrophysiological assessments. Lower boundary reference values for the HRUS data were obtained from literature. However, these values could only be applied to seven of our measurement sites, as the anatomical locations of the remaining nerve segments investigated differed from the reference papers [23, 24]. Additionally, detailed GAA repeat length data were missing in 8 of the 20 patients, and clinical severity was assessed solely using the SARA score.

## 5 Conclusion

The nerve morphology in FRDA showed variable nerve enlargement affecting nerves of the upper and lower extremities, and indicated a potential for nerve atrophy with or without prior nerve segment enlargement. No correlation was found between nerve enlargement and disease duration or severity. The cause of this sonographic pattern remains unclear. Further studies in larger patient cohorts are needed to reproduce our findings, clarify the underlying pathomechanism, and guide potential treatment.

## Author Contributions

K.K.: formal analysis (lead), writing – original draft (lead), writing – review and editing (equal). J.H.S.: writing – review and editing (equal). N.W.: writing – review and editing (equal). J.W.: writing – review and editing (equal). S.M.: writing – review and editing (equal). T.G.: writing – review and editing (equal). L.S.: writing – review and editing (equal). Z.F.: writing – review and editing (equal). L.S.: writing – review and editing (equal). S.H.: data collection, writing – review and editing (equal). A.G.: conceptualization (lead), writing – review and editing (equal).

## Funding

This work was supported by the Open Access Publishing Fund of the University of Tübingen.

## Ethics Statement

We confirm that we have read the journal's position on issues involved in ethical publication and affirm that this report is consistent with those guidelines.

## Consent

Consent was obtained from every patient before clinical and electrophysiological examinations.

## Conflicts of Interest

The authors declare no conflicts of interest.

## Supporting information


**Figure S1:** Schematic illustration of the examined nerves and the measurement points.

## Data Availability

The data that support the findings of this study are available from the corresponding author upon reasonable request.
