# Supplementary material for: Nerve Ultrasound in Patients With Friedreich Ataxia
Source: Muscle Nerve. 2025 Dec 4;73(3):395–402. doi: 10.1002/mus.70091 (PMC12888831; doi:10.1002/mus.70091)

Supplementary figure 1: Schematic illustration of the examined nerves and the measurement points

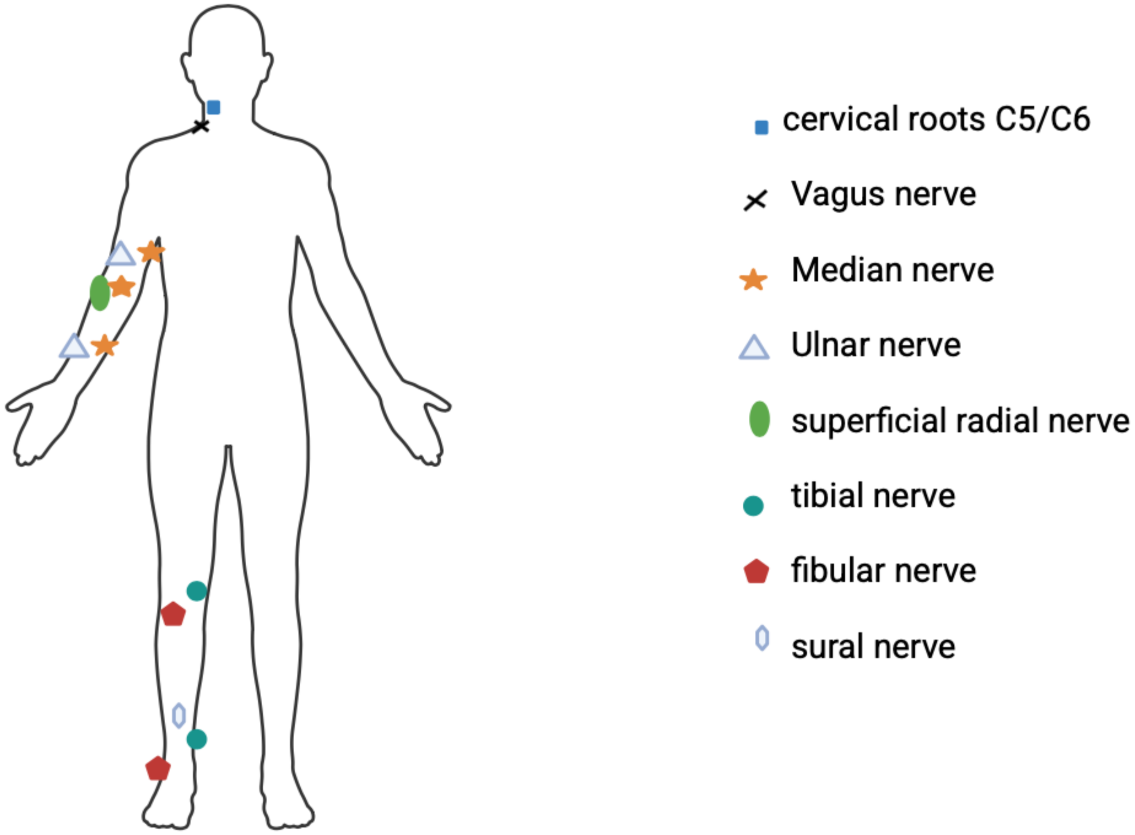

Supplement: Supplementary file 1 — Figure S1: Schematic illustration of the examined nerves and the measurement points. [file MUS-73-395-s001.pdf]
